# Intimate partner femicide, understanding perpetrator behaviour: a cross-cultural analytical review of weapon choice, injury patterns, and cultural-criminological insights

**DOI:** 10.1007/s00414-026-03843-9

**Published:** 2026-05-28

**Authors:** Dalila Tripi, Alessandro Ghamlouch, Fabio Del Duca, Maura Racciatti, Paola Frati, Stefano Ferracuti, Aniello Maiese

**Affiliations:** 1 Department of Anatomical, Histological, Forensic and Orthopaedic Sciences, Sapienza University of Rome, Viale Regina Elena 336, Rome, 00161 Italy; 2 Department of Human Neurosciences, Faculty of Medicine and Dentistry, Sapienza University of Rome, Rome, Italy; 3 Department of Biomedicine and Prevention, University of Rome “Tor Vergata”, Via Montpellier 1, Rome, 00133 Italy

## Abstract

Intimate Partner Femicide (IPF) is the lethal result of prolonged gender-based violence, often driven by control, jealousy, and emotional instability, while psychiatric disorders or substance abuse are reported in only a minority of cases. Victims typically endure years of abuse, facing significant barriers to escape due to fear, isolation, or dependency. Unlike IPF, Non-Intimate Partner Femicide (NIPF) involves perpetrators without close ties to the victim. Cultural and regional factors influence the occurrence and handling of femicides.

The aim of this narrative review with systematic literature search is to synthesize the epidemiological, criminological, and forensic patterns of intimate partner femicide (IPF) across different countries, focusing on victim and perpetrator profiles, common autopsy findings, risk factors, and cultural variations. Following the PRISMA statement, sixteen studies were retrieved on the epidemiology and criminology of femicide across several countries. In the U.S., most IPF cases involve prior abuse, with firearms being the most common weapon. Strangulation, blunt-force trauma, and poisoning are more common in low-middle income countries, where stricter gun laws exist. Psychiatric issues and substance abuse, particularly alcohol, are major risk factors, with mental disorders affecting 5–10% of perpetrators and 15–20% struggling with chronic substance use. Risk factors for IPF include young women (30–50), often employed, killed by long-term partners, with prior violence. The risk is highest after a relationship ends, especially in the first year. Violence often occurs in the victim’s home, where the abuser asserts dominance. Jealousy is a key motivator, escalating to violence as a tool of control, and is often seen as justified in patriarchal societies. The review stresses the need for prevention strategies that address these risks, including better mental health care, substance abuse treatment, and support for victims. Autopsy findings highlight common injuries in femicide cases, such as stab wounds, head injuries, and defensive wounds. These injuries, combined with other violent methods like strangulation, indicate an escalation of violence and are key to understanding femicide dynamics. The study emphasizes the importance of continued research and data collection to improve prevention and support systems for victims, aiming to reduce IPF rates globally.

## Introduction

Intimate Partner Femicide (IPF) is an extreme form of domestic violence that occurs when a man kills his current or former female partner [1, 2]. The most common forms of violence experienced by women are perpetrated by intimate partners [3–6]. The gendered nature of intimate partner femicide (IPF) has drawn particular attention [7]. Reducing violence against women has been recognized as a significant public health objective, integrated into international prevention strategies, policies, and programs [8].

Gender-based violence is closely linked to femicide and is often associated with physical, psychological, or sexual abuse [4]. Common criminological theories address key factors such as pre-existing relationships, prior abuse, control and possessiveness, and the final stage of escalating violence [9–13].

The perpetrator is often the victim’s current or former intimate partner, with whom she had a close relationship (e.g., married, cohabiting, or dating) [14–19]. In many cases, intimate partner femicide occurs after years of psychological, physical, or economic abuse, often ignored or minimized by those around the victim [15–22]. Pathological jealousy [16] and controlling behaviors [2] are common traits among perpetrators. Violent men seek to exert power over their partners and react violently to a perceived loss of control (e.g., at the end of the relationship) [2, 23]. 

Homicide typically represents the culmination of violence that has escalated over time. Many IPF victims had experienced previous assaults but were unable to escape or seek help due to fear, economic dependence, or social isolation [21]. 

Other types of gender-related violence are also examined in criminological research. In cases of non-intimate partner femicide (NIPF) [24], the perpetrator is someone who has no romantic or intimate relationship with the victim. This could be a stranger or someone with whom the victim had a superficial connection, such as an acquaintance, colleague, or friend, without lasting emotional ties.

Unlike intimate partner femicides, these killings are not always driven by dynamics of control or possessiveness [25]. In some cases, the perpetrator may be sexually motivated, such as in murders following rape, or driven by personal grudges, misogyny, or untreated psychological trauma [26–32]. They are often characterized by violent impulses, anger, or a desire for revenge. Some women are killed in the context of sexual violence or in circumstances where the perpetrator has no emotional connection to the victim [8, 33]. 

The connection between prevalence and psychopathology in IPF is essential to understanding the complexity of this phenomenon. On one hand, the high incidence of these homicides is closely linked to the presence of psychiatric risk factors in perpetrators, including personality disorders, substance abuse, and severe psychopathologies [31, 34]. On the other hand, the analysis of the pathological characteristics of both victims and perpetrators allows for the development of a criminological and forensic profile useful for prevention [2].

According to the literature, most victims had previously experienced psychological, physical, or economic violence, often downplayed or ignored [2]. Perpetrators, on the other hand, frequently exhibit controlling behaviors, pathological jealousy, and emotional instability, which can escalate into lethal violence [35]. Understanding these pathological aspects, alongside epidemiological data on IPF prevalence, enables the identification of women at risk and the development of more effective intervention strategies [8, 33, 36]. 

Violence and violent behaviors seem to vary across regions, possibly due to cultural differences and the social interpretation of women’s roles. The primary objective of this article is to describe the dominant demographic characteristics of IPF victims in the various countries analyzed and to provide recurrent patterns and shared risk characteristics among IPF victims. Secondly, it aims to examine criminological, forensic, and pathological features observed during autopsies. Finally, it seeks to identify the forensic psychiatric profiles associated with IPF perpetrators [36]. 

## Materials and Methods

**Fig. 1:**
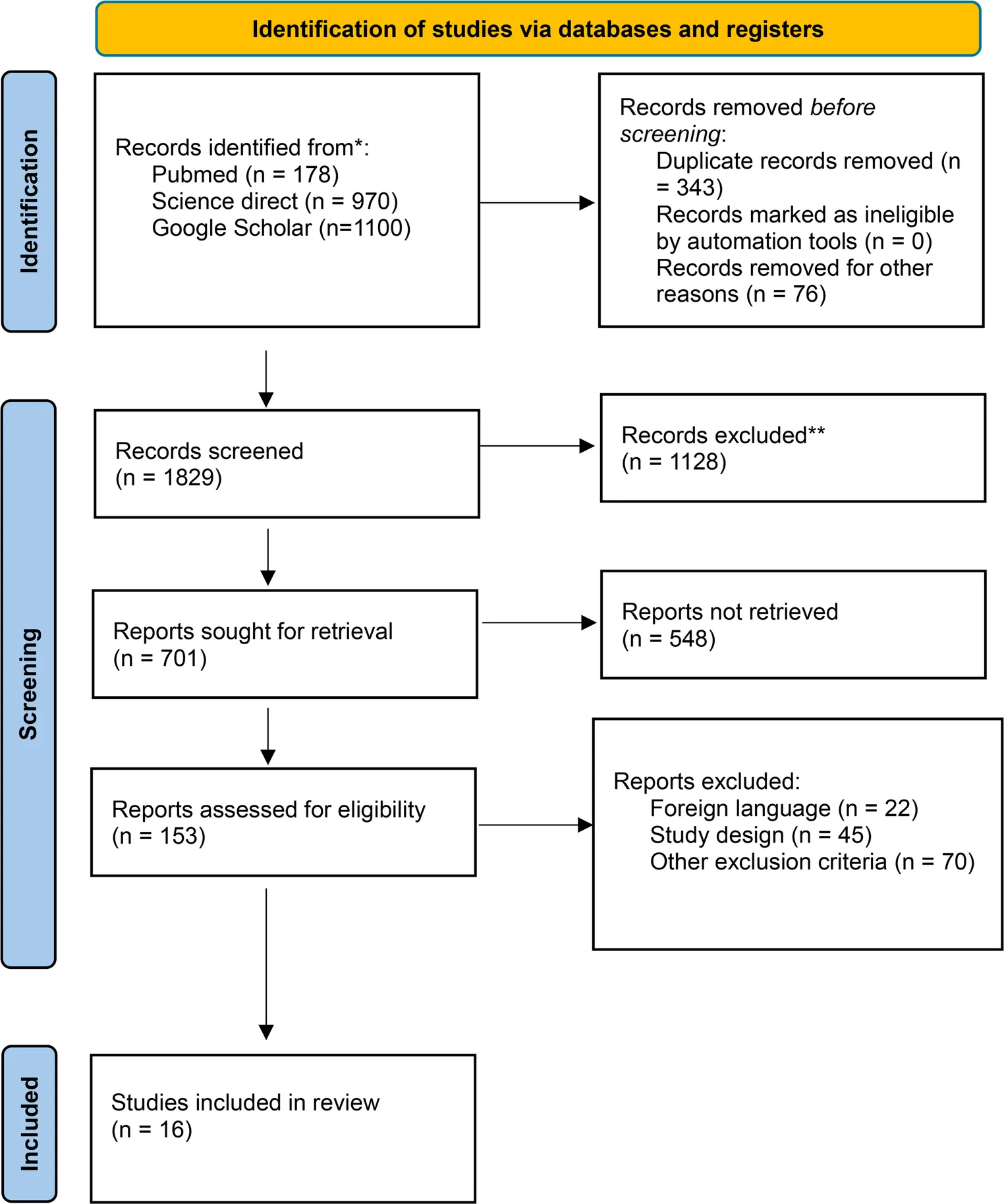
The selection of papers following PRISMA protocol

This narrative review with systematic literature search was conducted according to the PRISMA (Preferred Reporting Items for Systematic Reviews and Meta-Analyses) standards. A methodological assessment of each study was carried out following PRISMA guidelines, including an evaluation of bias. The PRISMA 2020 statement was applied, which consists of a checklist and a flow diagram (Fig. 1).

A narrative review with systematic literature search of the English-language literature on femicide and its forensic implications was conducted, followed by a critical review of the collected studies. The databases consulted included PubMed (178), Science Direct (970), Google Scholar (1100), and Scopus (92) from database inception to November 2024, using the following search terms:

(“Femicide” OR “intimate partner femicide” OR “IPF” OR “intimate partner homicide”) AND (“autopsy” OR “postmortem” OR “forensic” OR “post-mortem”).

For this review, the selection was limited to English-language studies to maintain methodological consistency and facilitate comparison between sources, given the greater availability of studies in this language within major scientific databases.

From this research, a dataset of abstracts was compiled, downloaded as a .nbib file, and uploaded into Zotero 6-0.30, used as a reference manager. The research group, following a dedicated meeting, established the inclusion and exclusion criteria for the selection of studies, in accordance with PRISMA standards [7]. 

Inclusion criteria were as follows:

1. Fully available publications in English;
2. Retrospective studies conducted on IPF victims subjected to autopsy examinations;
3. Retrospective studies focusing on the forensic psychiatric aspects of IPF perpetrators;
4. Demographic characteristics of the victims;
5. Demographic characteristics of the perpetrators;
6. Context of the homicide;
7. Victim-perpetrator relationship;
8. Perpetrators’ psychiatric history;
9. Motive for the homicide;
10. Geographical location of the event.

This study provides a valuable overview of common and divergent patterns found in femicide perpetrators worldwide. Due to substantial methodological heterogeneity and the descriptive nature of most studies, no formal quality assessment tool or meta-analysis was performed; study quality was evaluated narratively.

A large proportion of the initially identified records was excluded during the screening and eligibility phases, as shown in Fig. 1. In particular, most records were excluded during the title and abstract screening as they did not specifically address intimate partner femicide or lacked forensic, autopsy, or criminological data relevant to the aims of the review. At the full-text stage, further exclusions were applied based on the predefined inclusion criteria, particularly regarding the study design, availability of detailed victim–perpetrator relationship data, and the presence of psychiatric or forensic variables. Additional exclusions concerned non-English publications and limited accessibility of full-text articles. The final selection includes 16 studies providing sufficiently detailed and comparable data for the purposes of this analysis.

## Results

We conducted a systematic review of the scientific literature on the epidemiology and criminology of femicide for comparative analysis. Following the PRISMA-guided selection process (Fig. 1), sixteen retrospective studies were included in the final analysis. The main characteristics of the included studies—namely country of origin, study period, study design, sample size, availability of autopsy data, and psychiatric or criminological variables—are summarized in Table 1. The studies were published between 2004 and 2024 and covered a wide geographical distribution, including North America, Europe, South America, the Middle East, Asia, and Africa. Although all included articles were published within this time frame, several studies analyzed long retrospective series with observation periods extending back to the mid-20th century. Study designs were heterogeneous and included population-based registries, forensic autopsy series, and retrospective analyses of medico-legal or judicial records [37, 38].

**Table 1:** The studies were published between 2004 and 2024 and covered a wide geographical distribution

Author (year)	Country	Study period	Study design	IPF / NIPF	Sample size	Autopsy data	Psychiatric / criminological data	Main findings	Key limitations
Belfrage & Rying (2004)	Sweden	1990–1999	National retrospective	IPF	175	Partial	Yes	IPF associated with prior violence and jealousy	Registry-based data, limited psych detail
Henry (2010)	Alaska (USA)	1990–2008	Retrospective	IPF / sex-related	64	Partial	Yes	Firearms predominant	Small sample
Dobash & Dobash (2011)	UK	1990–2008	Qualitative / case review	IPF	122	No	Yes	Control and jealousy central	Narrative design
Kivivuori & Lehti (2012)	Finland	1990–2009	Population-based	IPF / other homicide	189	No	Limited	IPF differs from other homicides in relational dynamics	Lack of autopsy detail
Liem et al. (2013)	Finland, NL, Sweden	2003–2006	Multinational registry	IPF	577	No	Limited	Firearms less prevalent in Nordic countries	Heterogeneous datasets
Fong et al. (2016)	Taiwan	2003–2013	Autopsy-based	IPF	153	Yes	Limited	Sharp-force most frequent	Single-country
Meneghel et al. (2017)	Brazil	2003–2007	Population-based	IPF	521	No	Limited	High alcohol involvement	Underreporting
Toprak & Ersoy (2017)	Turkey	2000–2010	Registry-based	IPF	129	No	Limited	Strangulation and beating common	Media-based bias
Vatnar et al. (2017)	Norway	1990–2012	Retrospective forensic	IPF	177	Partial	Yes	Separation as major risk factor	Retrospective design
Salameh et al. (2018)	Jordan	2005–2015	Autopsy-based	IPF / NIPF	102	Yes	No	Sharp-force and strangulation prevalent	Limited psych data
Cunha & Gonçalves (2019)	Portugal	2000–2014	Retrospective	IPF	89	No	Yes	Substance abuse and prior convictions	Small sample
Zara et al. (2019)	Italy	1993–2013	Retrospective forensic	IPF / NIPF	113	Yes	Yes	Distinct criminological profiles	Regional data
Macucha & Taunde (2020)	Mozambique	2016–2018	Autopsy-based	IPF / NIPF	81	Yes	No	Non-partner killings prevalent	Short study period
Bologna study (2022)	Italy	1950–2020	Autopsy-based	IPF	276	Yes	Limited	Long-term trend analysis	Historical heterogeneity
Franchetti et al. (2024)	Germany / Italy	2000–2020	Autopsy-based	IPF	131	Yes	Partial	Injury patterns and weapon use	Forensic focus only
Lewis et al. (2024)	USA	2003–2020	National surveillance	IPF	> 2000	No	Yes	High prevalence of firearms and prior DV	Registry-based

Across the included studies focusing on intimate partner femicide (IPF), the perpetrator was most frequently identified as the victim’s current or former intimate partner, including husbands, cohabiting partners, or long-term romantic partners. In high-income countries population-based and registry studies, intimate partners accounted for approximately 75–80% of femicide perpetrators, with limited variability across countries [8, 39, 40]. In contrast, a different distribution was reported in specific regional contexts, such as Mozambique, where non-intimate perpetrators accounted for up to 76% of cases [41]. 

When the setting of the homicide was reported, the event most commonly occurred in a domestic environment, including the victim’s home or a shared residence. Population-based studies from the United States described a broader distribution of homicide locations, including public spaces and vehicles, although domestic settings remained predominant [42]. 

Marked geographical variability was observed in the methods of killing. In studies conducted in the United States and other high income countries, firearms represented the most frequently reported cause of death, accounting for approximately 40–50% of intimate partner femicides, followed by sharp-force injuries such as stabbing or cutting [37, 42]. In contrast, studies from low–middle income countries reported higher frequencies of strangulation, blunt-force trauma, and poisoning. In Mozambique and Turkey, strangulation accounted for approximately 25–30% of reported cases [41, 43]. Asian forensic autopsy series identified sharp-force injuries as the leading cause of death, followed by strangulation and physical beating [44, 45].

Several autopsy-based studies reported the presence of multiple injury mechanisms in the same victim, including combinations of strangulation, blunt-force trauma, and sharp-force injuries, reflecting complex assault dynamics [46, 47]. Defensive injuries, such as abrasions, contusions, and fractures of the upper limbs, were frequently described when detailed forensic examinations were available.

Information on psychiatric and criminological characteristics of perpetrators was variably reported across the included studies. Severe mental disorders, including psychotic disorders, were documented in approximately 5–10% of perpetrators in studies providing psychiatric data [34, 48]. A broader category of psychiatric symptomatology at the time of the offense, including depressive or psychotic features, was reported in up to 20% of cases in some forensic and registry-based studies [8]. Substance abuse emerged as a recurrent finding across multiple geographical areas. Chronic alcohol or drug abuse was reported in approximately 15–20% of perpetrators overall, with alcohol being the most frequently involved substance, followed by illicit drugs such as cocaine, methamphetamines, marijuana, and heroin [49]. Higher proportions of perpetrators with positive toxicological findings or documented substance abuse histories were reported in specific national studies, including South Africa, Portugal, England, and Brazil, with rates ranging between 30% and 40% [48, 49].

Several studies examined temporal and relational dynamics preceding the homicide. When data were available, the period following relationship separation or termination emerged as a particularly high-risk interval. In cases involving former partners, a substantial proportion of intimate partner femicides occurred within the first year after separation, with some studies reporting rates exceeding 50% [40, 50].

Due to substantial heterogeneity in study design, populations, outcome definitions, and data sources, no formal quantitative synthesis or meta-analysis was performed. All numerical values presented represent descriptive ranges derived from specific subsets of studies, including population-based registries, forensic autopsy series, or national reports, rather than pooled global estimates.

## Discussion

The findings of this review highlight the presence of recurrent criminological and forensic features in intimate partner femicide (IPF) across different geographical contexts. Although the included studies are heterogeneous in design and scope, several converging patterns emerge from the comparative analysis, particularly with regard to victim–perpetrator relationships, contextual dynamics of the homicide, and selected psychiatric risk factors [37, 51, 52].

The data do not support the identification of intimate partner femicide as a distinct legal category of homicide; however, they suggest that IPF is characterized by specific relational and contextual elements that differentiate it from other forms of lethal violence. From a medico-legal perspective, the recognition of these recurring features may contribute to improved risk assessment, preventive strategies, and interpretative frameworks in both forensic and judicial settings [36, 51].

Across the included studies, victims were most frequently adult women involved in long-term intimate relationships, often characterized by prior interpersonal violence and shared domestic or familial contexts. Perpetrators were predominantly current or former cohabiting partners, with limited regional variability reported in this regard. These observations, derived from population-based and forensic series, underscore the relevance of relational dynamics in the analysis of IPF cases [49, 52].

Several studies further identified the period following relationship separation as a particularly vulnerable phase, during which the risk of lethal escalation appears increased. In cases involving former partners, a substantial proportion of homicides occurred within the first year after separation, highlighting the temporal dimension of risk in IPF and its relevance for preventive and protective interventions [50, 52].

### Methods of killing and weapon availability

Across the included studies, marked geographical differences were observed in the methods used in intimate partner femicide (IPF). In population-based and registry studies conducted in high income countries, particularly in the United States and Western Europe, firearms emerged as the most frequently used weapon in IPF cases, whereas sharp-force injuries represented the second most common category [37, 42, 51].

In contrast, forensic and registry-based studies from low–middle income countries reported a higher prevalence of strangulation, blunt-force trauma, and sharp-force injuries, with firearms being less frequently involved [41, 43, 45].

These differences appear to be primarily related to variability in weapon availability and accessibility across regions. In countries with widespread civilian access to firearms, the presence of guns in domestic environments may facilitate their use during episodes of intimate partner violence, whereas in settings where firearms are less accessible, perpetrators more frequently resort to weapons readily available in the household, such as knives or to manual methods such as strangulation [37, 44]. From a medico-legal perspective, the method of killing has relevant implications for both injury patterns and investigative reconstruction. Autopsy-based studies consistently reported that strangulation and blunt-force trauma were frequently associated with multiple injury mechanisms and defensive wounds, reflecting intense physical confrontation between victim and perpetrator [46, 47].While sociocultural factors may contribute to shaping patterns of violence, the available empirical data primarily support an interpretation centered on structural differences in weapon accessibility rather than on specific cultural or symbolic meanings attributed to the act itself. Accordingly, explanations based on broader cultural or normative frameworks should be considered cautiously and remain secondary to the forensic and epidemiological evidence provided by the included studies.

### Setting

Across the included studies, the domestic environment emerged as the most frequent setting for intimate partner femicides. Population-based and forensic series consistently reported that the majority of IPF cases occurred in the victim’s home or in a shared residence with the perpetrator, whereas a smaller proportion took place in public spaces or other locations [42, 51, 52].

The predominance of the domestic setting appears to be closely related to the relational nature of IPF. Several studies described a background of prolonged intimate relationships characterized by prior interpersonal violence, shared living arrangements, and progressive escalation of abusive behaviors, which may facilitate lethal events within the domestic environment [37, 49].

From a forensic and medico-legal perspective, the domestic setting has relevant implications for both risk assessment and investigative reconstruction. Autopsy-based studies frequently reported evidence of prolonged physical confrontation and defensive injuries in cases occurring within the home, suggesting intense interpersonal dynamics preceding the fatal event [46].

Although theoretical models of coercive control have been proposed to explain the centrality of the domestic environment in intimate partner violence, the empirical findings of the included studies primarily support a descriptive interpretation centered on relational proximity, shared living spaces, and opportunity structures. Accordingly, broader psychological or sociological interpretations should be considered complementary to, but not substitutes for, the forensic and epidemiological evidence.

### Prior domestic violence and escalation patterns

Several of the included studies reported a documented history of prior interpersonal violence preceding intimate partner femicide. In population-based and forensic series, victims had frequently experienced repeated episodes of physical, psychological, or combined forms of abuse before the lethal event, suggesting a progressive escalation of violence over time [37, 51, 52].

When reported, prior violence often occurred within the context of long-term intimate relationships characterized by cohabitation and shared domestic environments. These relational and contextual elements may contribute to the persistence and intensification of abusive behaviors, ultimately increasing the risk of lethal outcomes [42, 49].

From a medico-legal perspective, the presence of a documented history of domestic violence has relevant implications for both risk assessment and prevention. Several studies emphasized that prior episodes of abuse, when identifiable through medical records, police reports, or forensic documentation, represent critical warning signals that may precede intimate partner femicide [51, 52].

While theoretical models describe domestic violence as a dynamic process involving escalation and increasing control, the empirical evidence provided by the included studies primarily supports a descriptive association between repeated prior abuse and the occurrence of IPF. Accordingly, interpretations extending beyond documented patterns of prior violence should be framed cautiously and distinguished from the observed data.

### Jealousy as a relational motive: medico-legal considerations

Several of the included studies identified jealousy and possessive behaviors as recurrent relational factors in cases of intimate partner femicide. In these contexts, jealousy was most frequently described as a motive reported in judicial records, witness statements, or case reconstructions, rather than as a formally diagnosed psychiatric condition [36, 51].

Importantly, the available evidence does not support the interpretation of jealousy as an independent psychopathological entity in the majority of IPF cases. Instead, jealousy appears to function as a relational and behavioral pattern, often embedded within dynamics of control, possessiveness, and fear of abandonment, particularly in the context of relationship conflict or separation [49, 52].

From a medico-legal perspective, this distinction is crucial. While pathological forms of jealousy may occur within specific psychiatric disorders (e.g., psychotic or substance-induced conditions), most cases described in the included studies involved non-delusional jealousy, expressed through controlling behaviors, threats, or escalating interpersonal violence. As such, jealousy should be interpreted primarily as a contextual risk factor rather than as a determinant of criminal responsibility.

Several studies emphasized that jealousy-related behaviors frequently intensified during periods of relational instability, particularly following separation or perceived threats to the relationship. In this phase, jealousy may interact with other risk factors—such as prior domestic violence, substance abuse, and access to weapons—contributing to an increased risk of lethal escalation [50, 52].

Accordingly, the relevance of jealousy in intimate partner femicide lies less in its psychiatric classification and more in its value as a warning signal within risk assessment frameworks. From a forensic standpoint, the identification of jealousy-driven behaviors in medical, legal, or social records may contribute to the early recognition of high-risk situations, without implying the presence of a mental disorder.

### Psychiatric disorders and substance abuse: forensic implications

Across the included studies, psychiatric disorders were reported in a minority of perpetrators of intimate partner femicide. When available, data indicated that severe mental disorders, including psychotic disorders, were present in approximately 5–10% of cases, whereas broader psychiatric symptomatology at the time of the offense—such as depressive or anxiety symptoms—was described in a higher but still limited proportion of perpetrators [34, 48, 51].

These findings suggest that most perpetrators of IPF do not meet criteria for severe mental illness and that psychiatric disorders alone cannot account for the occurrence of lethal violence. Rather, mental health conditions appear to function as contributing or amplifying factors when combined with relational conflict, prior domestic violence, substance abuse, and situational stressors, particularly during periods of relationship instability.

Substance abuse emerged as a more consistently reported factor across multiple studies. Chronic alcohol or drug abuse was documented in approximately 15–20% of perpetrators overall, with higher rates reported in specific national series. Alcohol was the most frequently involved substance, followed by illicit drugs such as cocaine, methamphetamines, marijuana, and heroin [49, 51]. Toxicological positivity at the time of the offense or a documented history of substance abuse was associated with increased impulsivity and reduced inhibitory control, potentially facilitating violent escalation.

From a medico-legal perspective, the presence of psychiatric disorders or substance abuse should therefore be interpreted within a multifactorial framework. While specific psychiatric conditions—particularly psychotic disorders or substance-induced states—may be relevant for individual assessments of criminal responsibility, the empirical evidence provided by the included studies does not support a general causal attribution of IPF to mental illness.

Accordingly, the forensic relevance of psychiatric factors in IPF lies primarily in their contribution to risk assessment, prevention strategies, and individualized medico-legal evaluation, rather than in their use as explanatory models for the phenomenon as a whole.

### Autopsy

There is a clear connection between the injuries observed during autopsies in femicide cases and the dynamics of domestic violence, coercive control, and the psychology of the perpetrator. Autopsies in these cases often reveal violent methods such as strangulation, stabbing, blunt force trauma, and sexual abuse, all of which are key indicators of an escalation of violence that frequently culminates in homicide.

These injuries, when analyzed in conjunction with cultural and social factors, provide valuable insights into understanding the phenomenon of femicide on an international level [4, 50–53]. 

Here are some indicative percentages based on global studies and reports on femicides:

1. Firearms: Firearms are used in a variable percentage of femicides, but they are generally a common weapon in intimate partner femicides (IPF). In countries where firearms are easily accessible, particularly in high-income countries [i.e. United States of America], the majority (45.2%) of femicides are committed using firearms.
2. Strangulation: Strangulation is one of the most common methods (27.1%) of femicides in low-middle income countries with restrictions on firearm access. It is followed by blunt force trauma (25.0%) and poisoning (16.7%).
3. Stab Wounds: Stab wounds are frequently observed in femicides, especially in countries where firearms are less accessible. Around 25–30% of intimate partner homicides involve the use of knives or similar sharp objects.
4. Head Injuries and Contusions: Head injuries and cranial trauma are very common in femicide cases related to domestic violence. Around 20–30% of intimate partner homicides involve head injuries, including skull fractures, brain hemorrhages, hematomas, and visible bruising. These are often associated with violent beatings or blunt force impacts.
5. Defensive Wounds: Defensive wounds (bruises, abrasions, or fractures resulting from the victim’s attempts to protect themselves) are common in femicides, as victims often try to resist or escape. On average, 25–30% of victims show defensive wounds, which are indicators of a struggle between the victim and the aggressor before the death.
6. Combination of Injuries: In many femicide cases, there is a combination of different types of injuries, suggesting an escalation of violence. This could include a mixture of strangulation, blunt force trauma, and stab wounds, indicating the aggressor’s increasing aggression .

**Fig. 2:**
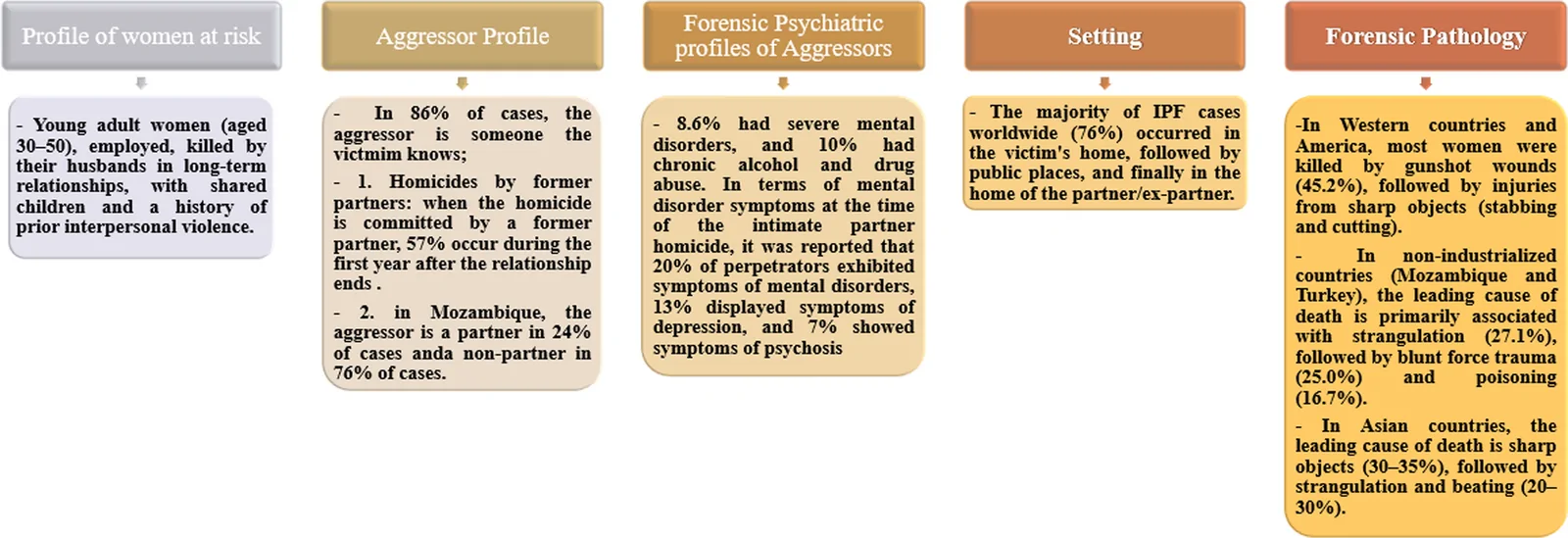
Diagnosis of femicide should derive from a global consideration of each feature discussed above, and reported in Fig. 1

The percentages presented are general estimates based on various international studies. It is important to note that specific data may vary between countries and contexts, influenced by factors such as weapon availability, domestic violence culture, and access to support resources for victims. However, strangulation, stab wounds, head trauma, and defensive injuries are the most commonly observed types of injuries during autopsies in femicide cases. These data are essential for understanding the dynamics of domestic violence and developing preventive strategies to reduce femicides globally [46, 47, 54–56] (Fig. 2). 

## Conclusion

In conclusion, the data analyzed in this study reveal a profile of women at risk, a profile of aggressors, and a common context at the international level. There is no international consensus on the causes of death in femicide cases, and this discrepancy is likely due to various cultural, social, and structural factors. However, empirical knowledge regarding intimate partner femicides (IPF), specific to the victims, is crucial for expanding the understanding of this phenomenon and for the development of effective prevention policies and strategies targeted at women at risk of IPF.

By identifying key risk factors, understanding the behavioral dynamics of aggressors, and considering cultural and social contexts, it becomes possible to design targeted interventions that can protect women and reduce the incidence of IPF globally. The ongoing collection of data, as well as the implementation of support systems for victims, are essential for building comprehensive.
